# Influence of Pre-Strain on the Course of Energy Dissipation and Durability in Low-Cycle Fatigue

**DOI:** 10.3390/ma18040893

**Published:** 2025-02-18

**Authors:** Stanisław Mroziński, Michał Piotrowski, Władysław Egner, Halina Egner

**Affiliations:** 1 Faculty of Mechanical Engineering, Bydgoszcz University of Science and Technology, Al. Kaliskiego 7, 85-796 Bydgoszcz, Poland; stanislaw.mrozinski@pbs.edu.pl (S.M.); michal.piotrowski@pbs.edu.pl (M.P.); 2 Faculty of Mechanical Engineering, Cracow University of Technology, Al. Jana Pawla II 37, 31-864 Kraków, Poland; wladyslaw.egner@pk.edu.pl

**Keywords:** low-cycle fatigue, dissipated energy, durability, hysteresis loop, pre-strain

## Abstract

The work undertaken in this paper is the comparative analysis of the accumulation of plastic strain energy in the as-received and pre-deformed (overloaded) material states, performed on the example of S420M steel. For this reason, the low-cycle fatigue tests on S420M steel specimens were conducted under controlled deformation conditions, and both as-received (undeformed) and pre-deformed specimens were used in the tests. The results of the low-cycle tests were analyzed in terms of dissipated energy. This study found that pre-straining of S420M steel specimens causes a reduction in the energy of the hysteresis loop at all strain amplitude levels. This results in a slight increase in the fatigue life of pre-strained specimens compared to as-received specimens. Based on the analysis, it was also found that despite the different lifetimes obtained at the same strain amplitude levels, the fatigue characteristics in terms of energy of the as-received and pre-strained samples are statistically the same. Experimental verification of the analytical models used to describe hysteresis loops confirmed their suitability for describing fatigue behavior for specimens made of steel in both the as-received and pre-strained state.

## 1. Introduction

Fatigue calculations of structural components are inextricably linked to the summation of fatigue damage. The basis for conducting calculations is knowledge of the fatigue characteristics (fatigue diagram) and load history. Depending on the description adopted of the fatigue process, the fatigue diagram can be presented in stress, strain or energy terms. The stress-based approach is recommended for so-called high-cycle fatigue, in which plastic deformation may occur only locally (e.g., in notched areas). The strain-based description is recommended when the applied loads cause plastic deformation (low-cycle fatigue). However, experimental tests [1] revealed the influence of the testing conditions on the hysteresis loop characteristics usually used to estimate the cumulative damage level in steels that do not exhibit a stabilization period during fatigue. The Ramberg–Osgood stress–strain curves for strain-controlled fatigue tests do not coincide with curves obtained at constant stress amplitude, while the differences are more pronounced at elevated temperatures. On the other hand, the total dissipated energy does not exhibit sensitivity to test conditions, due to the fact that the energy description takes into account both plastic strain and stress, and their mutual interaction. For this reason, the energy-based approach, greatly developed in recent years, can be used for fatigue calculations in both low-cycle and high-cycle fatigue cases. An extensive review of research on the energy-based description of the fatigue of materials was provided in [2].

Practical use of energy-based descriptions of the fatigue process sometimes requires a lot of auxiliary data obtained during low-cycle or static tests. Relationships have long been sought between the energy $U_{tf}$ dissipated until fracture during the monotonic test, the energy of a single hysteresis loop ${∆W}_{pl}$ and the total energy dissipated during cyclic loading $\Sigma {∆W}_{pl}(N_{f})$ until fracture. It is now known that the total energy dissipated in a variable-loaded sample, $\Sigma {∆W}_{pl}(N_{f})$ , is larger than the energy $U_{tf}$ dissipated during a monotonic test (cf. [3]). In addition, the dissipated energy in a variably loaded specimen until its fracture depends on the level of stress or strain, cf. [4,5,6,7]. Many works have confirmed the possibility of formulating close relationships between the unit energy of the hysteresis loop ${∆W}_{pl}$ or the cumulative energy $\Sigma {∆W}_{pl}(N_{f})$ and the durability $N_{f}$ , cf. [6,7,8]. These relationships have been applied to new methods of fatigue damage summation in terms of energy, cf. [9,10,11,12,13]. Detailed analyses of fatigue damage summation methods was carried out by [14,15].

The problem undertaken in this paper is the comparative analysis of energy dissipation in as-received and pre-deformed (overloaded) states, performed on the example of S420M steel under fatigue conditions. Pre-deformation of many technical objects such as nuclear power plants, thermal power plants, bridges and wind power plants, for example, can arise both during operation and before the period of their proper use. For example, they can be subjected to static loads resulting from improper commissioning, installation or, for example, shutdown due to seismic events. Such loads may result in irreversible plastic deformation of the structure occurring in some cases even before the period of use. Accumulation of the energy that is dissipated both before the period of use and during the actual period of operation can cause premature damage to a structural element that may lead to its fracture. The effects of unforeseen events causing pre-deformation are not usually taken into account in fatigue life prediction methods, which are most often based on material characteristics determined using materials in the as-received state. Due to the significant influence of pre-strains on durability, many scientific papers have been devoted to this topic. However, most of those analyses have been carried out using the strain description [16,17,18,19,20] or stress description [21,22,23,24]. For the reasons mentioned above, such studies do not allow general conclusions to be formulated. Therefore, the following research is aimed at clarifying the processes of damage accumulation in terms of energy dissipated due to pre-strains. The analysis presented in the paper is motivated by the investigation of the causes of a real existing bridge failure. After about 10 years of use, permanent deformation of welded attachment nodes was observed in the attachment areas of the bridge suspension cables. The magnitude of the observed deformations (almost 10%) made it necessary to close the bridge. Before deciding on a repair method for the bridge, a number of research works were carried out. One of them is the present work, the purpose of which was to determine the effect of pre-strain on the fatigue life of specimens made of S420M steel.

## 2. Materials and Methods

### 2.1. Description of Energy Accumulation

#### 2.1.1. Basic Assumptions

The basis of most energy descriptions of the fatigue process is the energy dissipated in the material during alternating loading until component failure, cf. [25]. It is assumed that the failure criterion is met when reaching the critical value of this energy. In most damage summation hypotheses, it is usually assumed that the unit energy of the hysteresis loop ${∆W}_{pl}$ does not change during the fatigue process. This means that the total energy dissipated during testing can be determined by multiplying the energy of the single loop in the so-called stabilization state by the number of fatigue cycles to fracture, $N_{f}$ :

(1) $$\Sigma \Delta W_{pl}\left(N_{f}\right)=N_{f}\cdot \Delta W_{pl}.$$

However, for many materials, the stabilized state does not occur or occurs for a very short period [4,5,6,7]. Therefore, the actual value of the cumulative energy $\Sigma {∆W}_{pl}(n)$ in terms of cyclic elastic–plastic deformation should be calculated by summing subsequent hysteresis loop energies, $\Delta W_{pl(i)}$ , which, in the case of an as-received specimen, can be written as follows:

(2) $$\Sigma \Delta W_{pl}\left(n\right)=\sum_{i=1}^{n}\Delta W_{pl\left(i\right)}.$$

In the case of pre-strain, the energy $U_{t}$ dissipated during pre-loading should be additionally taken into account in Equation (2). The total dissipated energy until fracture in such case can be determined from the following relation:

(3) $$\Sigma \Delta W_{pl}\left(n\right)=U_{t}+\sum_{i=1}^{n}\Delta W_{pl\left(i\right)}.$$

**Figure 1:**
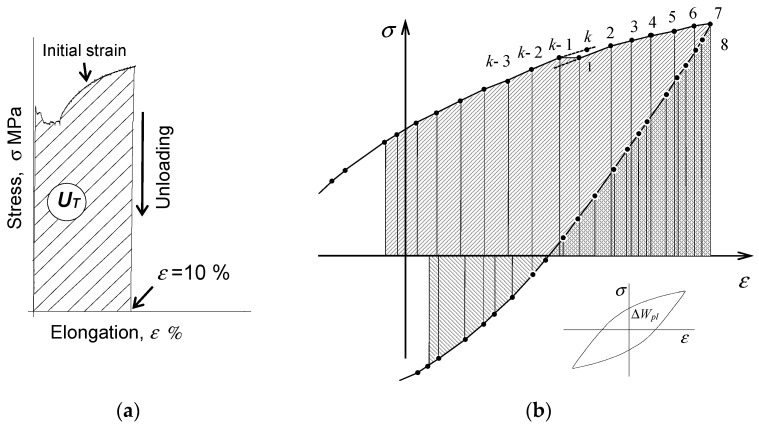
Interpretation of the dissipated energy during (**a**) initial deformation and (**b**) the fatigue test.

Figure 1 shows the value of energy $U_{t}$ obtained during the pre-strain stage. For the applied deformation level ( ε=10% ), it can be assumed that there is no change in the cross-section area of the specimen during pre-strain (the engineering stress has the same value as the true stress). Thus, the measure of energy dissipated during pre-strain, $U_{t}$ , is the area under the tensile diagram. This area can be determined from the following relation:

(4) $$U_{t}=\sum_{i=1}^{k}\frac{{(\sigma }_{i}+\sigma_{i+1})}{2}(\varepsilon_{i+1}-\varepsilon_{i}),$$

where $\sigma_{i}$ stands for the stress for the i -th force measurement, while $\varepsilon_{i}$ is the strain for the i -th measurement.

A measure of the unit energy ${∆W}_{pl}$ in one load cycle is the hysteresis loop area. Knowing the instantaneous values of the force loading the sample and its deformation, this energy can be calculated from the following relation:

(5) $$\Delta W_{pl}=\left[\sum_{i=1}^{k-1}\frac{1}{2}\left(\sigma_{i}+\sigma_{i+1}\right)\left(\varepsilon_{i+1}-\varepsilon_{i}\right)\right]+\left(\sigma_{k}+\sigma_{1}\right)\left(\varepsilon_{1}-\varepsilon_{k}\right),$$

where k —the number of measurements of the instantaneous value of the force loading the sample and its deformation during one loading cycle. Stress $\sigma_{i}$ is calculated as the ratio of the instantaneous value of the force loading the sample, $P_{i}$ , by the area of the initial cross-section of the sample, $S_{0}$ . The scheme for calculating the energy ${∆W}_{pl}$ under variable loading conditions is shown in Figure 1b.

The fatigue characteristics (fatigue diagram) in terms of critical single-loop energy is the relation $∆W_{pl}^{f}=f(N_{f})$ , which in the double logarithmic system can be described by a relation of the following form, cf. [10,13]:

(6) $$∆W_{pl}^{f}=\alpha_{w}\mathrm{lg}N_{f}+K_{w}.$$

To describe the critical total energy dissipation $\Sigma {∆W}_{pl}(N_{f})$ as a function of the number of cycles to fracture $N_{f}$ , one can use a similar equation of the form proposed in [4,10,13], among others:

(7) $$\Sigma \Delta W_{pl}(N_{f})=\alpha_{\Delta W}\mathrm{lg}N_{f}+K_{\Delta W}.$$

In paper [8], relation (7) was successfully used to describe the dissipated energy during variable loading of as-cast and austempered ductile irons. It was found that the relationship between energy $\Sigma {∆W}_{pl}(N_{f})$ and durability $N_{f}$ is an excellent material characteristic. It was shown in [26] that the free expression of Equation (7) for $N_{f}=0.25$ corresponds to the dissipated energy $U_{tf}$ during a static tensile test (specimens made of C45 structural steel were used during the tests). Static tensile tests were conducted while controlling the diameter of the loaded specimens. In [12], the graph described by Equation (7) was adopted as the basis in the damage summation hypothesis formulated for the energy-based description. Experimental verification of the hypothesis showed high agreement between fatigue life results from tests and calculations.

#### 2.1.2. Analytical Description of Hysteresis Loop

The basis for the descriptions of fatigue in terms of energy, presented in the previous section, was the result of experimental tests. These included, among other elements, hysteresis loops recorded during alternating loading at applied load levels. When predicting fatigue life in energy terms, we usually do not have source results in the form of recorded hysteresis loops. A criterion quantity such as the unit energy of the hysteresis loop ${∆W}_{pl}$ has to be calculated using material data obtained during low-cycle fatigue tests. This involves an analytical description of the relevant branches of the hysteresis loop. For this purpose, the relationship between stress amplitude $\sigma_{a}$ and total strain amplitude $\varepsilon_{at}$ is most often used in the form of the equation proposed in [27]:

(8) $$\varepsilon_{at}=\frac{\sigma_{a}}{E}+{\left(\frac{\sigma_{a}}{K^{\prime }}\right)}^{\frac{1}{n^{\prime }}},$$

where E is Young’s modulus, while $K^{\prime }$ and $n^{\prime }$ are other material parameters.

The equation of the increasing branch of the hysteresis loop is obtained by multiplying Equation (8) by two, which can be written as follows:

(9) $$∆\varepsilon =\frac{∆\sigma }{E}+2{\left(\frac{∆\sigma }{2K^{\prime }}\right)}^{\frac{1}{n^{\prime }}}.$$

**Figure 2:**
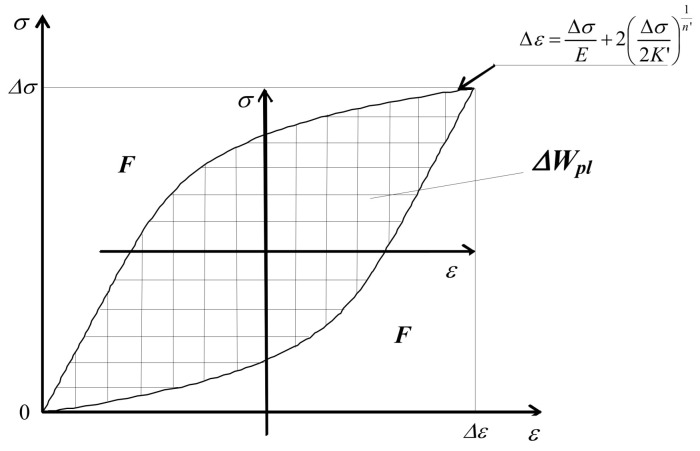
Diagram for calculating the energy of the hysteresis loop ${∆W}_{pl}$ .

The decreasing branches of the loop are obtained by applying Equation (9) after transforming the coordinate system to the upper vertex of the loop. The resulting formulas are used to describe hysteresis loops of materials subject to the so-called Masing rule [28]. For materials not subject to this rule, a so-called skeleton diagram is defined, which is formed by the upper or lower branches of the hysteresis loop, cf. [29]. Figure 2 shows the methodology for proceeding with the calculation of energy ${∆W}_{pl}$ for a material subject to Masing’s rule.

According to the scheme shown in Figure 2, the relationship for the unit energy ${∆W}_{pl}$ can be written as

(10) $$∆W_{pl}=∆\sigma ∆\varepsilon -2F=∆\sigma ∆\varepsilon -2\int_{0}^{∆\sigma }\left(\frac{∆\sigma }{E}+2{\left(\frac{∆\sigma }{2K^{\prime }}\right)}^{\frac{1}{n^{\prime }}}\right)d\sigma$$

and the final form of the equation for energy ${∆W}_{pl}$ can be written as

(11) $$∆W_{pl}=∆\sigma ∆\varepsilon -\frac{{∆\sigma }^{2}}{E}-4\frac{∆\sigma^{\frac{1}{n^{\prime }}+1}}{\left({\left(2K^{\prime }\right)}^{\frac{1}{n^{\prime }}}\right)\left(\frac{1}{n^{\prime }}+1\right)}.$$

### 2.2. Experimental Tests

#### 2.2.1. Static Tensile Tests

**Figure 3:**
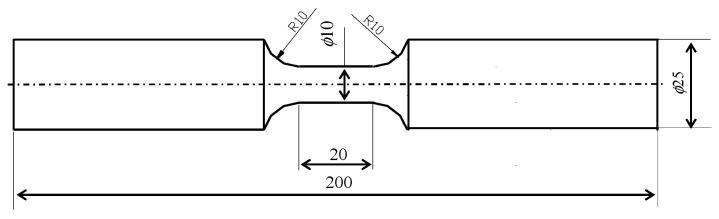
Test specimen.

The test specimens were made from S420M steel and shaped according to the guidelines specified in the standard in [30]. The chemical composition of the steel is given in Table 1, while the dimensions of the specimen are shown in Figure 3. The tests were carried out in accordance with the standard in [31]. During the tests, the instantaneous values of the loading force and elongation of the specimen were recorded. The elongation of the specimen was measured using a static test extensometer (type 2630-110) with a 10 mm base mounted on the measuring part of the specimen, and a measuring range of +100%. The force was measured using a force gauge head (2518-113) with a measuring range of ±100 kN. The tests were carried out at a temperature of ${21\;}^{o}C$ . The separation of the specimen in the measuring part was taken as the criterion for the end of the static test.

#### 2.2.2. Fatigue Tests

**Figure 4:**
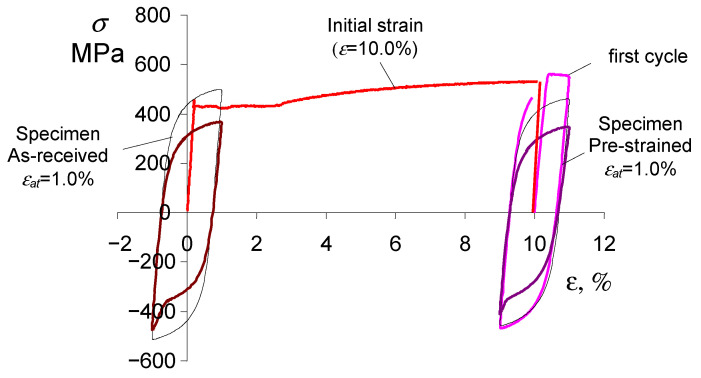
Fatigue testing methodology for as-received and pre-deformed specimens.

Fatigue tests were conducted using undeformed specimens (as-received) and specimens subjected to preliminary deformation ε=10% . The speed of the piston of the testing machine during preloading was the same as during static tensile testing, and was 0.05 mm/s. After unloading, the specimens were subjected to cyclic loading at five levels of controlled strain $\varepsilon_{at}=const$ ( $\varepsilon_{at}$ = 0.25%, 0.35%, 0.5%, 0.8%, 1.0%). Figure 4 shows schematically the methodology adopted for testing the as-delivered and pre-strained specimens.

Fatigue tests were carried out at room temperature on an Intron 8502 testing machine (High Wycombe, UK). The deformation of the specimen was measured with an extensometer with a measuring base of 10 mm. The loading frequency during the tests was 0.2 Hz. During the tests, the instantaneous values of the force loading the specimen and its deformation were recorded. The sampling frequency of the force and strain signal adopted during the tests made it possible to describe hysteresis loops with 200 points. As a criterion for the end of the fatigue tests, the appearance of a so-called kink in the hysteresis loop in the compression half-cycle was adopted (cf. [30]).

#### 2.2.3. Statistical Analysis

Three low-cycle fatigue tests at each strain level were implemented during the tests. When developing the results, the unit loop energy $∆W_{pl}$ and accumulated energy $\Sigma \Delta W_{pl}$ (an independent variable) were calculated individually for each specimen, based on the hysteresis loops obtained at different fatigue life periods. Thus, at each strain level, the energy values were characterized by a certain scatter, and each point of the graphs placed below had a different value of it. The spread of the results was determined by linear regression analysis, for which 95% confidence intervals were determined.

The tables included in the figures summarize the values of the parameters appearing in the regression equations and the values of the $R^{2}$ correlation coefficients.

## 3. Results

### 3.1. Static Tensile Tests

**Figure 5:**
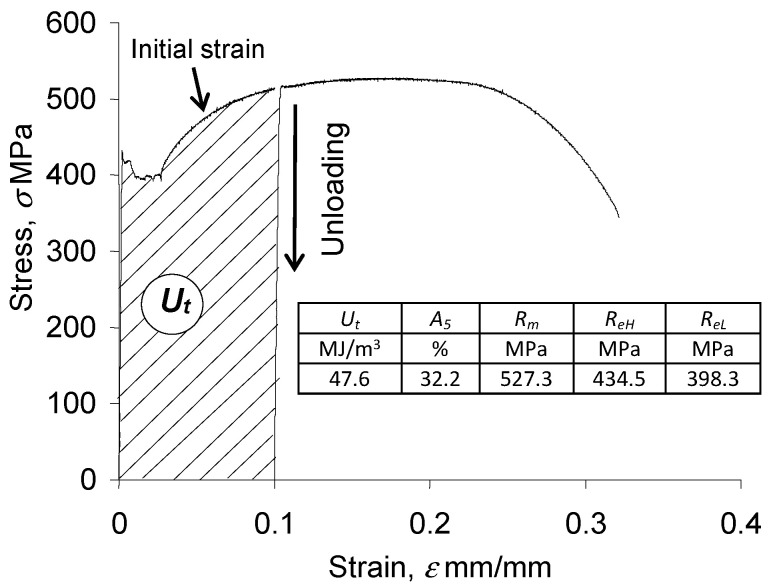
Monotonic tensile diagram.

Figure 5 shows an exemplary tensile diagram in the σ−ε coordinate system. The table summarizes the determined strength parameters of S420M steel and the energy $U_{t}$ dissipated during the initial deformation of 15 specimens.

### 3.2. Low-Cycle Fatigue Tests—Strain-Based Description

**Figure 6:**
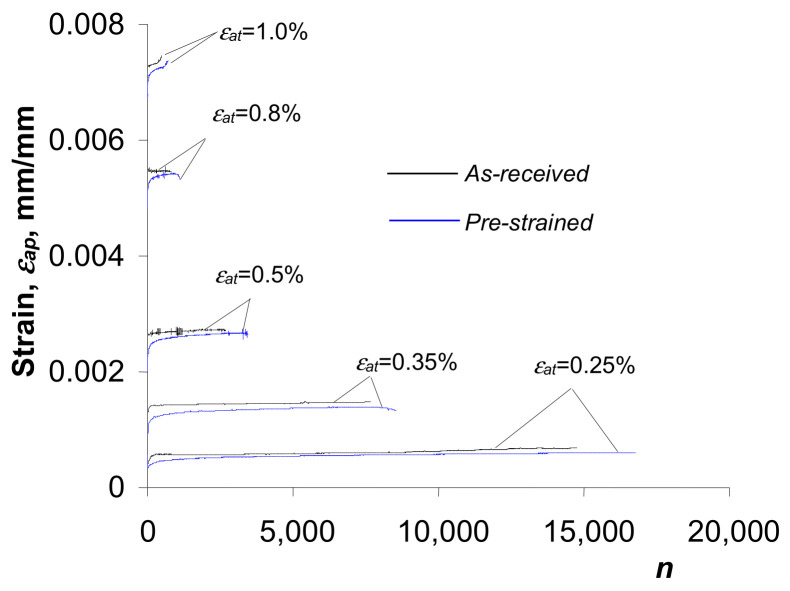
Plastic strain amplitude $\varepsilon_{ap}$ as a function of the number of cycles n .

The results of the low-cycle fatigue tests were developed in accordance with the procedures specified in the standard in [30]. Detailed test results for S420M steel specimens are provided in [20]. Therefore, the rest of this paper is limited only to those results that are closely related to the energy-based description of fatigue presented in Section 3.1. Figure 6 shows plastic strain amplitude $\varepsilon_{ap}$ as a function of the number of loading cycles n , for the as-received and pre-deformed specimens.

The analysis of the graphs (Figure 6) makes it possible to conclude that during cyclic loading, the plastic strain amplitude $\varepsilon_{ap}$ of the as-received specimens is larger than for the pre-strained specimens. This demonstrates the effect of pre-strain on the low-cycle properties of S420M steel. Comparative strain analysis of $\varepsilon_{ap}$ reveals that after permanent deformation ε=10% , S420M steel undergoes slight hardening. Due to the lack of a clear stabilization period for this steel (cf. [20]), the hysteresis loop parameters for further analysis were determined from a period corresponding to the half-life ( $n/N_{f}=0.5$ ), as it is commonly adopted in the case of non-stabilizing materials. To describe the analytical relationship between stress $\sigma_{a}$ and strain $\varepsilon_{ap}$ , an equation of the form proposed in [30] was adopted:

(12) $$lg\sigma_{a}={lgK}^{\prime }+n^{\prime }lg\varepsilon_{ap}.$$

**Figure 7:**
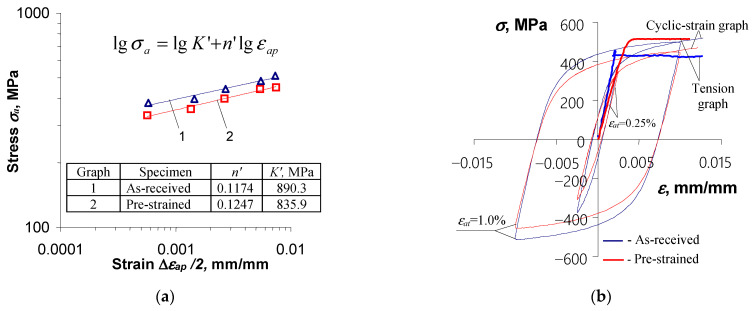
Results for as-received and pre-deformed specimens: (**a**) cyclic graphs and (**b**) cyclic and monotonic tension graphs.

Figure 7a shows the graphs described by Equation (12), while Figure 7b shows the graphs described by Equation (8).

**Figure 8:**
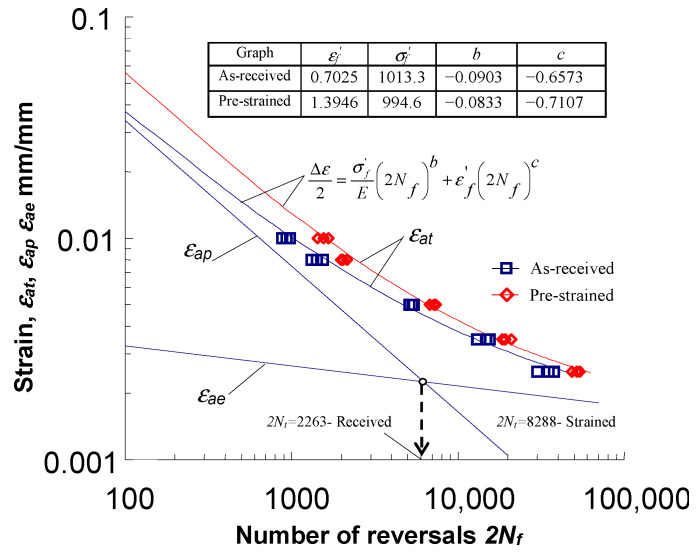
Fatigue life diagrams.

The fatigue life results are shown in Figure 8 in the $2N_{f}-\varepsilon$ coordinate system using the Manson equation (cf. [32]) of the following form:

(13) $$\frac{\Delta \varepsilon_{at}}{2}=\frac{\Delta \varepsilon_{ae}}{2}+\frac{\Delta \varepsilon_{ap}}{2}=\frac{\sigma_{f}^{\prime }}{E}{\left(2N_{f}\right)}^{b}+\varepsilon_{f}^{\prime }{\left(2N_{f}\right)}^{c}.$$

Based on the analysis of the results of the low-cycle fatigue tests, it can be concluded that at the same strain amplitude levels $\varepsilon_{at}$ the durability of the pre-strained specimens is slightly higher than that of the as-received specimens. The increase in durability is a consequence of the reduction in strain $\varepsilon_{ap}$ due to pre-strain (see Figure 6). The increase in the durability of pre-strained specimens is more pronounced at lower total strain amplitude levels $\varepsilon_{at}$ . As the strain increases, the difference in the obtained durability decreases. Experimental details of the effect of pre-strain on the durability of S420M steel specimens under controlled deformation conditions are described in [20].

### 3.3. Low-Cycle Fatigue Tests—Energy-Based Description

**Figure 9:**
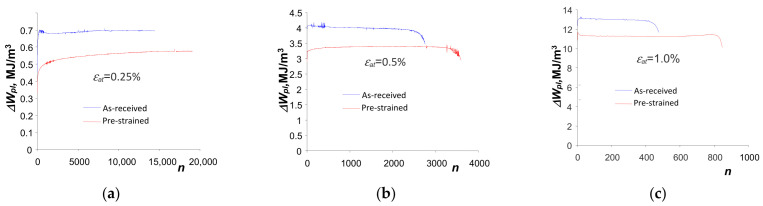
Unit loop energy ${∆W}_{pl}$ for specimens in as-received state and after initial deformation: (**a**) $\varepsilon_{at}$ = 0.25%, (**b**) $\varepsilon_{at}$ = 0.5%, (**c**) $\varepsilon_{at}$ = 1.0%.

The values of the loading force and deformation of the specimen recorded during the low-cycle fatigue tests for individual loading cycles were processed to determine the unit loop energy of plastic deformation, ${∆W}_{pl}$ . The unit energy calculations were carried out according to Equation (5), and the methodology shown in Figure 1b. Based on a comparative analysis of the plots of unit energy ${∆W}_{pl}$ as a function of the number of cycles n , it was found that the unit energy in pre-strained samples is always lower than the unit energy in samples in the as-received state. To illustrate this effect, Figure 9 shows the unit energy waveforms at three strain amplitude levels ( $\varepsilon_{at}$ = 0.25%, $\varepsilon_{at}$ = 0.5%, $\varepsilon_{at}$ = 1.0%).

**Figure 10:**
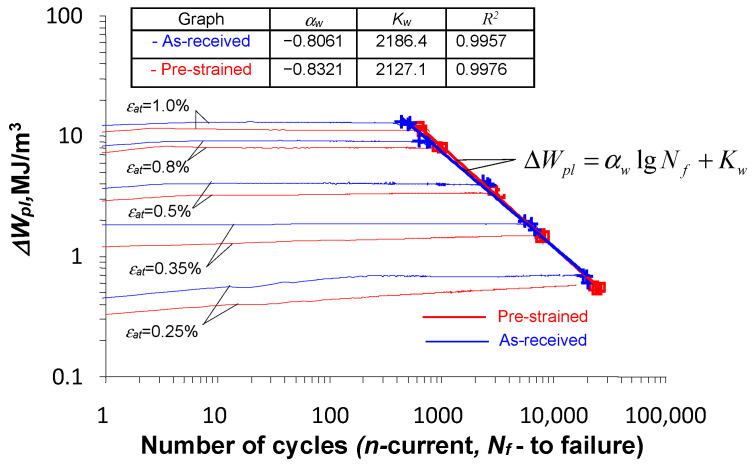
Unit energy ${∆W}_{pl}=f(N_{f})$ changes and fatigue diagrams.

Figure 10 presents the graphs of energy ${∆W}_{pl}$ as a function of the number of cycles n, for five levels of deformation. Marks (crosses and squares) indicate the obtained results for durability N . The fatigue diagrams ${∆W}_{pl}=f(N_{f})$ described by Equation (6) and the values of the directional coefficient $\alpha_{w}$ and the free expression $K_{w}$ of this equation are also shown in Figure 10.

**Figure 11:**
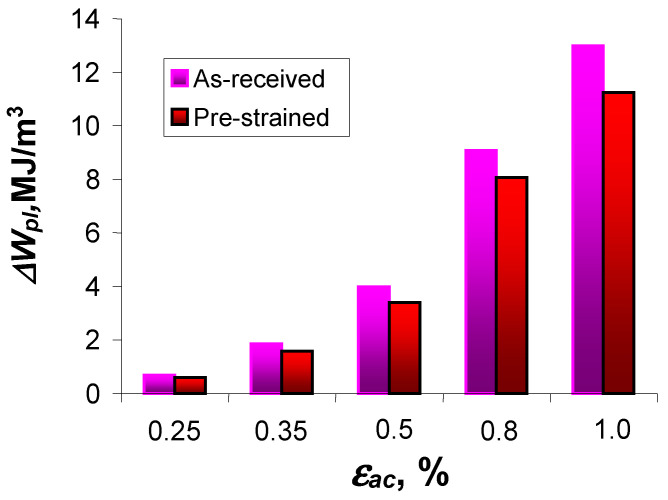
Unit energy ${∆W}_{pl}$ for samples in the as-received and pre-deformed states, at the life stage of $n/N_{f}=0.5$ .

Based on the analysis of the position of the fatigue diagrams in the $N-{∆W}_{pl}$ coordinate system, it can be concluded that they are characterized by only a slight variation. Confirmation that the differences are indeed small is provided by the similar values of the free expression $K_{w}$ and the directional coefficient $\alpha_{w}$ of the regression equation. Based on the analysis of the graphs, there was no difference statistically at the significance level of α=0.05 in the results of the fatigue life tests described by Equation (6) for the as-received and pre-strained specimens. The small changes in energy ${∆W}_{pl}$ as a function of the number of loading cycles confirm reports in the literature of the low sensitivity of this parameter to changes in cyclic properties, cf. [4,8,11,13]. The smaller range of plastic deformation ${∆\varepsilon }_{ap}$ and stress ∆σ after pre-straining (Figure 6) resulted in the unit energy ${∆W}_{pl}$ of the pre-strained specimens at all strain levels being lower than the unit energy obtained for specimens in the as-received state. To illustrate the differences, Figure 11 compares the energy ${∆W}_{pl}$ values corresponding to half of the fatigue life $n/N_{f}=0.5$ , at five strain levels, for the as-received and pre-strained samples.

**Figure 12:**
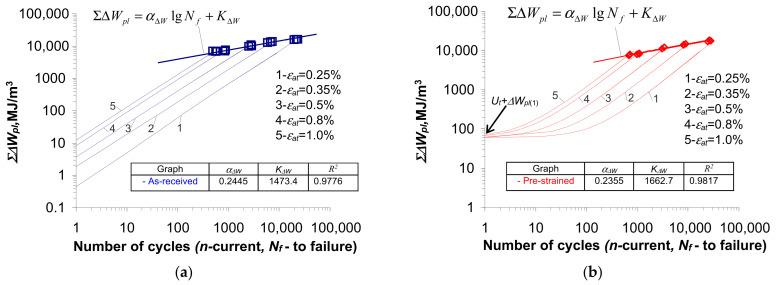
Energy accumulation $\Sigma {∆W}_{pl}$ : (**a**) samples in the as-received state and (**b**) pre-deformed samples.

Figure 12 presents, in a bi-logarithmic coordinate system, sample plots of loop energy accumulation at five levels of strain amplitude $\varepsilon_{at}$ for samples in the as-received state (Figure 12a) and pre-strained state (Figure 12b). According to the basic assumptions and Equation (3), the strain energy $U_{t}$ obtained during monotonic testing was taken into account when summing the energy dissipation of the pre-strained specimens (Figure 5). In Figure 12, the functions of the critical cumulative energy $\Sigma {∆W}_{pl}(N_{f})$ are also plotted, according to Equation (7).

**Figure 13:**
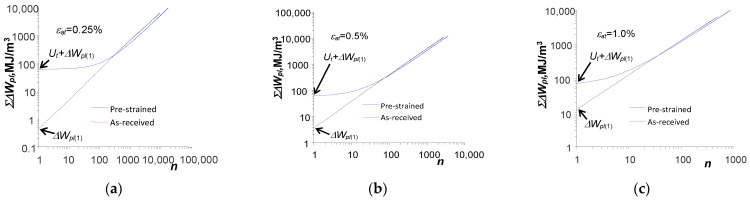
Unit energy accumulation in as-received and pre-strained samples: (**a**) $\varepsilon_{at}$ = 0.25%, (**b**) $\varepsilon_{at}$ = 0.5%, (**c**) $\varepsilon_{at}$ = 1.0%.

In order to better compare the course of energy accumulation in the as-received samples and pre-deformed samples, Figure 13 shows the graphs of accumulation at three chosen strain levels, i.e., $\varepsilon_{at}$ = 0.25%, $\varepsilon_{at}$ = 0.5% and $\varepsilon_{at}$ = 1.0%.

Based on the analysis of the graphs shown in Figure 12a and Figure 13, it can be concluded that small changes in energy ${∆W}_{pl}(n)$ for the samples in the as-received state (Figure 9 and Figure 10) result in a linear bi-logarithmic pattern. However, in the case of pre-strained specimens (Figure 12b and Figure 13), the linear nature of energy accumulation during the initial life period was disrupted by the pre-strain of the specimen.

**Figure 14:**
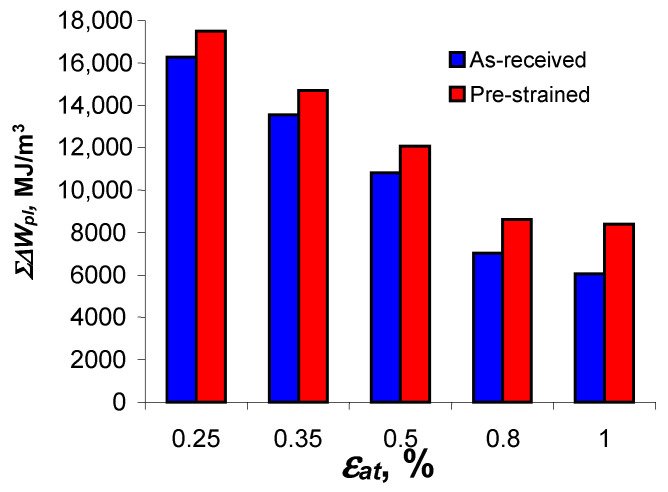
Accumulated energy $\Sigma {∆W}_{pl}(N)$ for pre-strained and as-received samples.

Based on the position of the graphs describing the values of cumulative energy $\Sigma {∆W}_{pl}(N_{f})$ in the as-received and pre-strained samples, it can be seen that these values are affected by the level of strain amplitude, $\varepsilon_{at}$ . The energy $\Sigma {∆W}_{pl}(N_{f})$ increases as the strain level $\varepsilon_{at}$ decreases. The above confirms the test results described in [4,8,12,13], among others. For direct comparison, Figure 14 shows graphs illustrating the values of critical cumulative energy (at fracture) for the as-received and pre-strained specimens.

At all levels of deformation, the values of critical cumulative energy $\Sigma {∆W}_{pl}(N_{f})$ for pre-strained specimens are larger than for as-received specimens, while the differences are affected by the level of deformation. The difference between the energies is smallest at the highest strain level $\varepsilon_{at}$ = 0.25%, (about 7%) and increases to about 25% at the lowest strain level $\varepsilon_{at}$ = 1.0%. Despite the different loading histories and the variation in cumulative energy at different strain levels in the specimens in the as-received and pre-deformed states, the obtained cumulative energies can be described by a simple regression rule (7).

**Figure 15:**
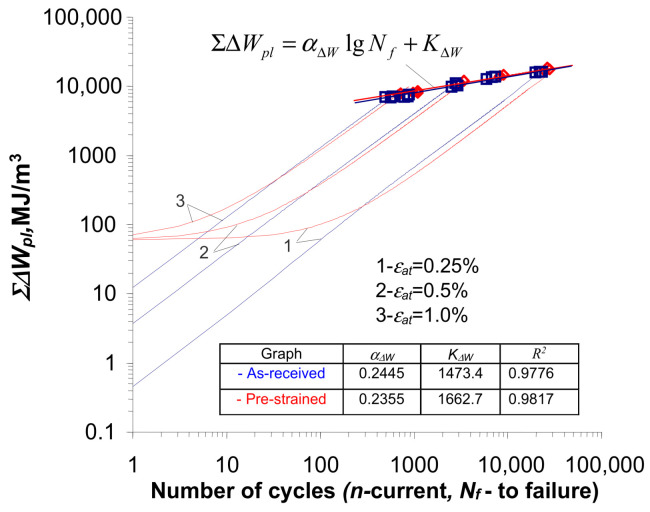
Energy accumulation in the as-received and pre-strained specimens.

In order to compare the course of cumulative unit energy in the samples in the as-received and pre-strained states, Figure 15 compares in one coordinate system the waveforms obtained for both cases.

For statistical evaluation, a parallelism test and a free expression test of the regression lines described by Equation (7) were carried out. It was found that there were no grounds to reject the null hypotheses of equality of regression coefficients $\alpha_{\Delta W}$ and free expression $K_{\Delta W}$ of both regression lines. The above confirms the results of the tests described in [4,8], where the relationship $\Sigma {∆W}_{pl}\left(N_{f}\right)=f(N_{f})$ (directional coefficient and free expression) was called the material parameter. It should be pointed out that, in the case of the regression lines for the $\Sigma \Delta W_{pl}$ and $\Delta W_{pl}$ , apart from the parallelism of the lines for the as-received and pre-strained samples, the lines and their corresponding confidence intervals practically coincide.

## 4. Discussion: Verification of Analytical Models

**Figure 16:**
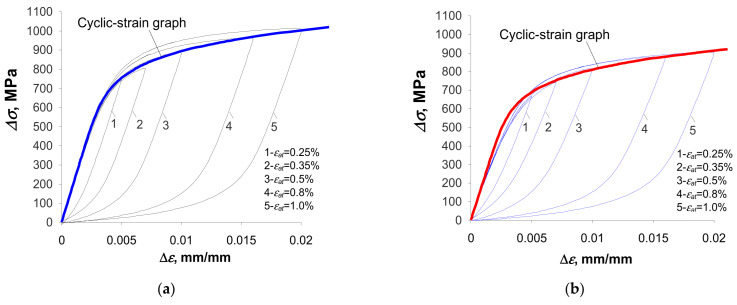
Hysteresis loops at the life stage $n/N_{f}=0.5$ : (**a**) samples in the as-received state and (**b**) pre-deformed samples.

Figure 16 shows the hysteresis loops for the period corresponding to half of the fatigue life ( $n/N_{f}=0.5$ ) for the samples in the as-received state and the pre-strained samples. To illustrate the quality of the representation of the branches of the hysteresis loops, the graph described by Equation (9) is also plotted in the figure.

Based on a comparative analysis of the graph described by Equation (9) and the growing branches of the experimental hysteresis loops, it can be concluded that the as-received samples do not fully exhibit behavior consistent with the Masing model. By subjecting the material to pre-straining, the growing branches of the experimental hysteresis loops (Figure 13b) better fit the shape described by Equation (9). In this regard, the results confirm the findings reported in [4,19]. Due to the relatively slight variation in the position of the graph describing the ascending branch of the loop in relation to the actual loops for both types of material, in the following work it was assumed that the S420M steel adopted for the study satisfies Masing’s principle, and further calculations of the energy ${∆W}_{pl}$ were carried out using Equation (11).

**Figure 17:**
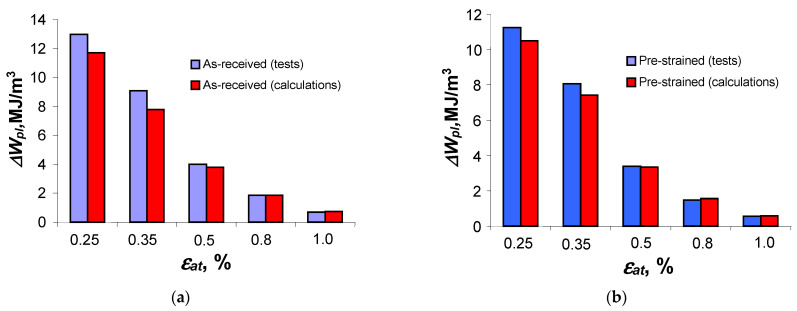
Loop energy ${∆W}_{pl}$ obtained from calculations and tests: (**a**) as-received samples and (**b**) pre-deformed samples.

The results of the hysteresis loop energy calculation, ${∆W}_{pl(C)}$ and the results of the loop energy from the tests, ${∆W}_{pl(E)}$ , for the as-received and pre-deformed specimens are summarized in Figure 17. The results refer to the value of the unit energy ${∆W}_{pl}$ at half fatigue life $n/N_{f}=0.5$ .

It can be concluded that for both the as-received and pre-deformed samples, the values of the calculated unit energy ${∆W}_{pl}$ are for most levels of deformation less than the energy obtained from testing. The situation changes at the lowest load levels where the unit energy from the calculations is larger than the loop energy from the tests.

**Figure 18:**
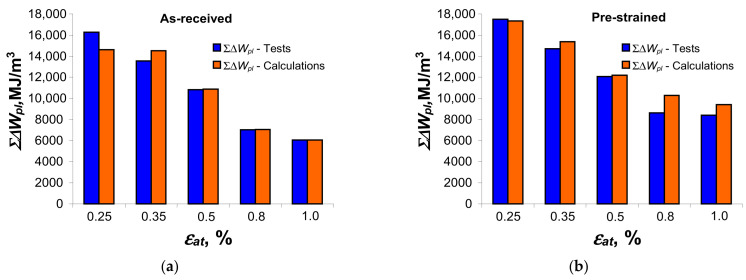
Cumulative energy during calculation and testing: (**a**) as-received samples and (**b**) pre-deformed samples.

The calculated values of the unit loop energy ${∆W}_{pl}$ corresponding to $n/N_{f}=0.5$ were used to evaluate the total dissipated energy $\Sigma {∆W}_{pl}(N_{f})$ during the fatigue test. Figure 18 compares the results of the calculation and testing of the total dissipated energy for the as-received specimen and the pre-strained specimen. When calculating the energy $\Sigma {∆W}_{pl}(N_{f})$ for the pre-strained specimens, the energy $U_{t}$ obtained during the pre-strain of the specimens was taken into account (see Figure 5).

**Figure 19:**
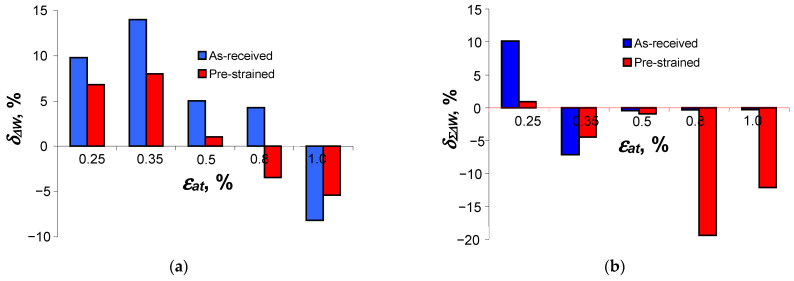
Mapping errors of (**a**) unit loop energy ${∆W}_{pl}$ and (**b**) cumulative energy $\Sigma {∆W}_{pl}$ .

The values of total cumulative energy obtained for the as-received and pre-deformed samples are very similar. In most cases, the cumulative energies $\Sigma {∆W}_{pl}(N_{f})$ calculated are slightly higher than those obtained during testing. In order to compare the effectiveness of the ${∆W}_{pl}$ and $\Sigma {∆W}_{pl}(N_{f})$ material data used for energy calculations, Figure 19 shows the mapping errors of the energy dissipated in one cycle, $\delta_{\Delta W}$ , and the energy dissipated during fatigue testing, $\delta_{\Sigma \Delta W}$ , calculated from the following relationships:

For unit energy ${∆W}_{pl}$ :

(14) $$\delta_{\Delta W}=\frac{\Delta W_{pl(E)}-\Delta W_{pl(C)}}{\Delta W_{pl(E)}}\cdot 100\%.$$

For cumulative energy $\Sigma {∆W}_{pl}$ :

(15) $$\delta_{\Sigma \Delta W}=\frac{\Sigma \Delta W_{pl(E)}-\Sigma \Delta W_{pl(C)}}{\Sigma \Delta W_{pl(E)}}\cdot 100\%.$$

where ${∆W}_{pl(E)}$ —loop energy from tests from period $n/N_{f}=0.5$ ; ${∆W}_{pl(C)}$ —loop energy from calculations; $\Sigma {∆W}_{pl(E)}$ —total dissipated energy obtained during testing; and $\Sigma {∆W}_{pl(C)}$ —total dissipated energy obtained during calculations.

A comparative analysis of the errors $\delta_{\Delta W}$ and $\delta_{\Sigma \Delta W}$ allows us to conclude that the use of material data for a material that better conforms to the Masing model (pre-strained samples) allows us to obtain smaller unit energy mapping errors. For the as-received specimens, the maximum loop energy mapping errors $\delta_{\Delta W}$ depend on the level of deformation, and amount to almost 15% for the level $\varepsilon_{at}$ = 0.35%. In contrast, for the pre-strained samples at the same level, the errors do not exceed 6%.

The small changes in the unit energy ${∆W}_{pl}$ as a function of the number of cycles mean that the cumulative energy $\Sigma {∆W}_{pl}(N_{f})$ , calculated for the as-received and pre-strained samples, differs only slightly. However, it should be underlined that the calculated values of energy ${∆W}_{pl}$ and cumulative energy $\Sigma {∆W}_{pl}(N_{f})$ , as well as the errors $\delta_{\Delta W}$ and $\delta_{\Sigma \Delta W}$ , apply only to the durability for which the parameters $n^{\prime }$ and $K^{\prime }$ ( $n/N_{f}=0.5$ ) were determined. Consequently, for durations $n/N_{f}<0.5$ and $n/N_{f}>0.5$ , the values of specific energy ${∆W}_{pl}$ , cumulative energy $\Sigma {∆W}_{pl}(N_{f})$ and the mapping errors of these parameters may differ from the values shown in Figure 19.

## 5. Conclusions

The study presented in this paper investigated the effect of pre-strain on the course of plastic strain energy accumulation in S420M steel specimens subjected to variable loading. For this purpose, low-cycle fatigue experimental tests were carried out on as-received specimens and specimens pre-strained to ε=10% . The experimental results were used to evaluate the course of unit loop energy accumulation, ${∆W}_{pl}$ . Based on this study, the following conclusions were made:

1. Total dissipated energy until fracture, $\Sigma {∆W}_{pl}(N)$ , depends on the level of strain amplitude $\varepsilon_{at}$ . The dissipated energy increases as the strain level decreases.
2. Unit loop energy, ${∆W}_{pl}$ , accounts for stresses, strains and their interactions; therefore, it is not sensitive to the type of control quantities (stresses or strains) used in experimental tests. It can be classified as a parameter that is not very sensitive to changes in the cyclic properties of steel, and the classical analytical descriptions are capable of reproducing well the experimental values of the unit loop energy of both as-received and pre-strained specimens.
3. The pre-straining of S420M steel specimens causes a reduction in the loop plastic strain ${\Delta \varepsilon }_{pl}$ . Thus, a decrease in the unit hysteresis loop energy ${∆W}_{pl}$ is observed at all levels of strain amplitude $\varepsilon_{at}$ . For this reason, a slight increase in the fatigue life of pre-strained specimens is observed compared to as-received specimens.
4. Despite the different durabilities $N_{f}$ obtained at the same strain levels $\varepsilon_{at}$ for the pre-strained and as-received samples, their fatigue characteristics in terms of energy, i.e., the fatigue plot ${∆W}_{pl}=f(N_{f})$ and the accumulation plot $\Sigma {∆W}_{pl}=f(N_{f})$ are statistically the same.
5. There is a close relationship between the total energy dissipated during fatigue testing $\Sigma {∆W}_{pl}$ and the durability $N_{f}$ . In fatigue calculations, the relationship $\Sigma {∆W}_{pl}=f(N_{f})$ can perform similar functions as the classical fatigue diagram of ${∆W}_{pl}=f(N_{f})$ . The parameters of Equation (7), i.e., the directional coefficient and the free expression, can be material-specific quantities (cf. [8]), and Mroziński and Topoliński [13] utilized the relation $\Sigma {∆W}_{pl}=f(N_{f})$ in a new method of fatigue damage summation in terms of energy, pointing out the advantages of the energy-based approach in degradation modeling, cf. [1].

## Figures and Tables

**Table 1:** Chemical composition of S420M steel (wt %).

Fe	C	Si	Mn	P	Cr	Al	Nb	Ti	V	W
98.0	0.125	0.215	1.45	0.0135	0.0208	0.0268	0.0288	0.013	0.0519	0.0150

## Data Availability

The original contributions presented in this study are included in the article. Further inquiries can be directed to the corresponding author.

## List of Symbols

E	Young’s modulus
$R_{m}$	tensile strength
$R_{eH}$	upper yield strength
$R_{eL}$	lower yield strength
$K^{\prime }$	cyclic strength coefficient
n	current number of cycles of constant-amplitude loading
$n^{\prime }$	cyclic strain-hardening exponent
$N_{f}$	number of cycles to failure
$2N_{f}$	number of reversals to failure
$2N_{t}$	transition life
$n/N_{f}$	relative number of cycles
$\alpha_{W}$ , $\alpha_{\Delta W}$	energy per cycle exponent
$K_{W}$ , $K_{\Delta W}$	energy per cycle coefficient
$\Delta W_{pl}$	plastic strain energy in a unit volume of material per cycle (area of the hysteresis loop)
$U_{t}$	energy dissipated during static pre-strain
$U_{tf}$	energy dissipated during static pre-strain until failure
σ	stress in general terms
Δσ	cyclic stress range
$\sigma_{a}$	amplitude of stress
ε	strain in general terms
$\varepsilon_{at}$	amplitude of total strain
$\varepsilon_{ap}$	amplitude of plastic strain
$\varepsilon_{ae}$	amplitude of elastic strain
Δε	cyclic total strain range
$\Delta \varepsilon_{ap}$	cyclic plastic strain range
$\varepsilon_{f}’$	coefficient of cyclic plastic strain
$\sigma_{f}’$	fatigue strength coefficient
b , c	exponents of strain diagram of fatigue
